# Abnormally decreased renal Klotho is linked to endoplasmic reticulum-associated degradation in mice

**DOI:** 10.7150/ijms.68137

**Published:** 2022-01-09

**Authors:** ShaSha Li, JiaWei Kong, LiXia Yu, QiFeng Liu

**Affiliations:** 1 Clinical Research & Lab Centre, Affiliated Kunshan Hospital of Jiangsu University, 91 Qianjin West Road, Kunshan, Jiangsu, 215300, China.; 2 Department of Nephrology, Affiliated Kunshan Hospital of Jiangsu University, 91 Qianjin West Road, Kunshan, Jiangsu, 215300, China.

**Keywords:** Klotho, unfolded protein response, endoplasmic reticulum-associated degradation, renal interstitial fibrosis

## Abstract

**Aim**: Endoplasmic reticulum-associated degradation (ERAD), which involves degradation of improperly folded proteins retained in the ER, is implicated in various diseases including chronic kidney disease. This study is aimed to determine the role of ERAD in Klotho deficiency of mice and human kidney tubular epithelial cells (HK-2) with renal interstitial fibrosis (RIF).

**Methods**: Following establishment of a mouse RIF model by unilateral ureteral obstruction (UUO), a specific ERAD inhibitor, Eeyarestatin I (EerI), was administered to experimental animals by intraperitoneal injection. Serum and kidney samples were collected for analysis 10 days after operation. Soluble Klotho levels were measured by enzyme-linked immunosorbent assay, while the degree of kidney injury was assessed by renal histopathology. Renal Klotho expression was determined by quantitative real-time PCR, immunohistochemical and western blotting analyses. ERAD and unfolded protein response (UPR) were evaluated by detecting associated components such as Derlin-1, glucose-regulated protein 78 (GRP78), activating transcription factor 4 (ATF4) and protein disulfide isomerase (PDI). HK-2 cells were exposed to transforming growth factor (TGF)-β1 with or without EerI, and expressions of related proteins including Klotho, Derlin-1, GRP78, ATF4 and PDI were determined by western blotting analyses.

**Results**: UUO induced severe kidney injuries and RIF. Klotho expression in both serum and kidney tissue was obviously downregulated, while Derlin-1 was notably upregulated, indicating that ERAD was activated to potentially degrade improperly folded Klotho protein in this model. Intriguingly, treatment with EerI led to significantly increased Klotho expression, especially soluble (functional) Klotho. Furthermore, specific inhibition of ERAD increased expression of GRP78, ATF4 and PDI compared with the UUO group. The consistent results *in vitro* were also obtained in TGF-β1-treated HK-2 cells exposed to EerI. These observations suggest that UPR was remarkably enhanced in the presence of ERAD inhibition and compensated for excess improperly folded proteins, subsequently contributing to the additional production of mature Klotho protein.

**Conclusion**: ERAD is involved in Klotho deficiency in RIF and its specific inhibition significantly promoted Klotho expression, possibly through enhanced UPR. This may represent a novel regulatory mechanism and new therapeutic target for reversing Klotho deficiency.

## Introduction

Klotho is a longevity gene with powerful kidney-protective effects such as anti-oxidation, anti-inflammation, anti-apoptosis, and suppression of renal fibrosis 1-4. Klotho exists as both membranous-bound (mKlotho) and soluble (sKlotho) forms. mKlotho is a type 1 transmembrane glycoprotein with a short intracellular domain, membrane spanning domain, and large extracellular domain. sKlotho is produced by ectodomain shed of mKlotho or alternative Klotho mRNA splicing 5. Because Klotho is mainly generated by the kidney, it is easily understood that Klotho expression is influenced by the state of kidney function 6, 7. Indeed, its expression is severely reduced in kidney diseases such as chronic kidney disease (CKD) 8 and acute kidney injury 9. Considering its beneficial effects on kidney, deficiency of Klotho in CKD inevitably makes the kidney vulnerable to suffer various insults, aggravates kidney function, and promotes CKD progression 10. In contrast, an increase in Klotho level can improve kidney function and arrest CKD progression 11. Therefore, upregulation of endogenous Klotho expression or reversing the decline of endogenous Klotho expression is a plausible therapeutic strategy to slow down CKD 12, 13. However, causes of Klotho deficiency in CKD remain unclear 14. Previous studies demonstrated that Klotho expression is severely suppressed at the transcript level in early or moderate CKD stages due to altered epigenetic modifications in the Klotho gene 15, 16. Yet, epigenetic modifications only partly explain the observed Klotho deficiency because correcting epigenetic abnormalities in the Klotho gene did not fully rescue Klotho expression 15, 16. Therefore, other regulatory mechanisms are implicated in Klotho deficiency and deserve comprehensive investigation.

CKD is characterized by increased endoplasmic reticulum (ER) stress and concomitant with Klotho deficiency, indicating potential interplay between ER stress and Klotho expression 8, 17-19. We previously observed that rats with unilateral ureter obstruction (UUO) induced-renal interstitial fibrosis (RIF) featured both ER stress ignition and Klotho loss, and Klotho replacement treatment repressed renal ER stress and improved their kidney function 20. Furthermore, a recent study demonstrated that inhibition of ER stress rescued Klotho protein downregulation in mice with proteinuria CKD 21, suggesting a close link between ER stress and Klotho expression 22. Persistent stress in CKD contributes to the continuous deposition of misfolded or unfolded proteins in the ER 23. As an adaptive mechanism, the unfolded protein response (UPR) activates to address these aberrant proteins or precursors retained in the ER and restore ER homeostasis 24. ER-associated degradation (ERAD), served as a critical branch of ER stress, is subsequently triggered to degrade these improperly folded proteins, thereby maintaining cellular homeostasis 25. Considering its critical physiological significance, ERAD is implicated in the pathogenesis and development of diverse diseases including kidney diseases 26-28. Hence, we speculate that CKD disturbs normal processing of Klotho protein, results in aggregation of improperly folded Klotho in ER, and consequently accelerates Klotho protein degradation via the ERAD pathway, thus contributing at least partially to Klotho deficiency.

Here, we assessed the impacts of ERAD on Klotho protein expression using canonical CKD models including UUO-induced RIF and transforming growth factor (TGF)-β1-treated human kidney tubular epithelial cells (HK-2). Our findings unraveled a novel modulatory mechanism of Klotho expression and potential therapeutic targets for the rescue of Klotho expression under CKD.

## Materials and methods

### Animals and experimental procedures

All experiments were performed in accordance with the guidelines for the Animal Care and Use Committee of Kunshan First People's Hospital. Male C57BL/6 mice (20-25 g, 8 weeks old), purchased from Joinn Laboratories Co. Ltd. (Suzhou, China), were free access to food and water in at light-, temperature- and humidity-controlled environment. All mice were subjected to either a sham or UUO operation and were assigned randomly to 4 groups (n = 6, each) as follows: sham, UUO, Eeyarestatin I (EerI) treatment and UUO plus EerI treatment. UUO procedure was performed as previously described 20. Briefly, experimental animals were anesthetized generally, and the right ureter was double-ligated at two locations following abdominal incision. Consequently, the right ureter was cut between the two locations. Mice in sham group undergone identical UUO procedure but the ureteral was not obstructed or ligated. EerI (Santa Cruz, sc-358130B), an ERAD inhibitor, was injected into abdominal cavity in UUO mice at a dosage of 2.5 mg/kg, and repeated on day. All mice were sacrificed at day 10 after UUO operation. Serum and kidney samples were collected and processed properly for further analyses.

### Histopathologic examination

Fixed renal tissues were prepared and embedded in paraffin. Embedded-paraffin kidneys were cut into 4 μm-thickness. Then, the cut sections were deparaffinized, rehydrated and subsequently stained with Hematoxylin and Eosin (H&E) as well as Masson trichrome using routine histologic methods. The degree of renal interstitial damage was examined by independent pathologists blinded to the group assignation under light microscope. RIF was calculated semiquantitatively from 10 randomly selected fields of each renal slide by evaluating blue-stained areas in Masson trichrome.

### RNA isolation and quantitative real-time PCR

Total RNA was exacted and purified by Tissue RNA Purification Kit Plus (Shanghai Yishan Biotechnology Co., LTD, #RN002plus), and cDNA was synthesized using EasyQuick RT MasterMix according to manufacturer's instructions (Cwbio, #CW2019M). PCR was carried out with UltraSYBR Mixture (Cwbio, # CW0957M) using the LightCycler 480 Ⅱ system (Roche). The cycling program was pre-denaturated at 95°C for 10 min, which was followed by 40 cycles of 10 s at 95°C, 30 s at 60°C and 30 s at 72°C. Relative *klotho* expression levels were measured using 2-^ΔΔCt^ method. The primers used in this study were listed as follows: *klotho*, forward 5'-GGCTTTCCTCCTTTACCTGAAAA-3', reverse 5'-CACATCCCACAGATAGACATTCG-3'; *GAPDH*, forward 5'-AACGACCCCTTCATTGAC-3', reverse 5'- TCCACGACATACTCAGCA-3'.

### Immunohistochemical analysis

Paraffin-bedded samples were dewaxed, rehydrated and then heated in citrated buffer for antigen retrieval. After washing with phosphate buffer saline (PBS), the slides were blocked in normal rabbit serum and incubated with primary anti-Klotho antibody (abcam, ab181373) at 4 ℃ overnight. Afterward, slides were washed by PBS and incubated with biotinylated secondary antibody for 1 h at room temperature. At last, the immunohistochemical reactions were visualized with diaminobenzidine and counterstained with hematoxylin.

### Measurement of sKlotho level

Mouse serum sKlotho level was determined by enzyme linked immunosorbent assay (ELISA) according to instructions. The kit was purchased from Elabscience Biotechnology Co., Ltd., which use the Sandwich-ELISA principle. The optical density (OD) is measured spectrophotometrically at the wavelength of 450 nm. The concentration of sKlotho was calculated by Origin 9.1 software according to the OD value of each sample. Theoretically, only mature Kotho type can be transferred from ER and located in the plasma membrane, otherwise it is degraded in the cytoplasm via ERAD and can't contribute to the generation of sKlotho via mKlotho ectodomain shedding. That's to say, sKlotho represents the mature and functional Klotho.

### Cell culture

Human proximal tubular epithelial cells HK-2 (National Collection of Authenticated Cell Cultures, Shanghai, China) were grown in DMEM/F12 with low glucose containing 10% fetal bovine serum in a humidified atmosphere of 5% CO_2_ at 37 °C. To investigate the effect of EerI on renal fibrosis, HK-2 cells were exposed to TGF-β1 at the dosage of 10 ng/mL (one of the most potent fibrogenic cytokines) in presence or absence of EerI (10 μM). After 24 h, cells were harvested for western blotting analyses.

### Western blotting analyses

Frozen renal tissues and harvested cells were homogenized in Radio-Immunoprecipitation Assay (RIPA) lysis buffer containing protease inhibitor cocktail. After centrifuged at 12,000 rpm for 15 min at 4 ℃, and the protein concentration of the supernatant was measured using the BCA protein assay kit (Thermo Fisher, No. 23227). 20 μg of total protein were fractioned by 8% or 10% SDS-polyacrylamide gel electrophrosis and transferred to polyvinylidene fluoride membranes. After blocking with 5% non-fat milk for 1 h at room temperature, the membranes were incubated with anti-Klotho (abcam, ab181373; Santa Cruz, sc-515942), glucose regulated protein 78 (GRP78) (Abcam, ab21685), activating transcription factor 4 (ATF4) (Santa Cruz, sc-390063), protein disulfide isomerase (PDI) (CST, #3501), Derlin-1 (Abcam, ab176732), α-smooth muscle actin (α-SMA) (CST, #14968), E-cadherin (E-cad) (CST, #14472), β-actin (Santa Cruz, sc-8432), GAPDH (Abcam, ab181603) antibodies, overnight at 4°C, and subsequently incubated with HRP-conjugated secondary antibodies for 1 h at room temperature. The expression levels were measured and quantified using Image J software and then normalized to β-actin or GAPDH.

### Statistical analysis

The original data are presented as mean ± standard error (SD). Data analysis is carried out via one-way analysis of variance (ANOVA) followed by Student-Newman-Keuls (SNK) test among groups. Significant difference among groups is set as p value < 0.05.

## Results

### Establishment of UUO-induced RIF

**Figure 1:**
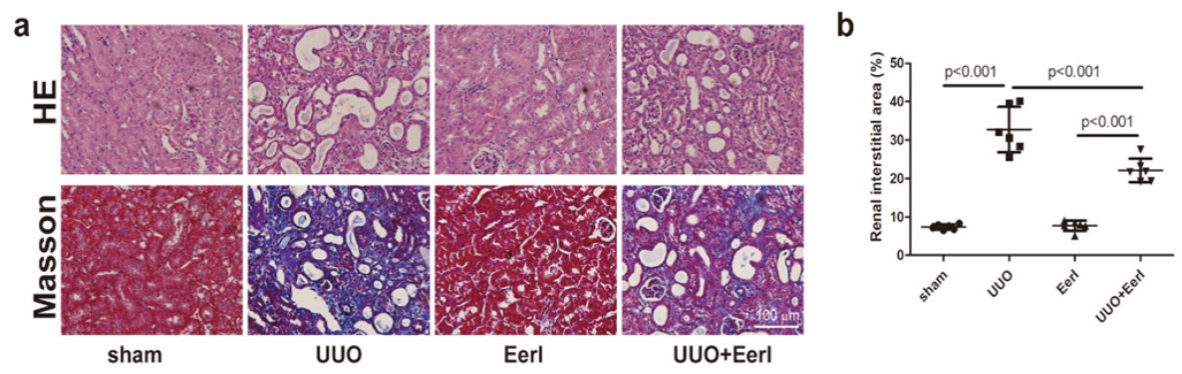
** EerI alleviates renal fibrosis induced by UUO in mice.** (a) Representative micrographs of HE and Masson trichrome (scale bar, 100 μm). (b) Semiquantitative data of fibrosis area. Data are presented as the mean ± SD (n = 6).

Mice were sacrificed 10 days after operation. Characteristic pathological lesions from kidney samples subjected to UUO surgery, including kidney tubular dilation, epithelial cell necrosis, renal interstitial edema, and inflammatory cell infiltration, were observed by H&E staining (Figure 1a). In addition, with respect to RIF induced by UUO, severe RIF was also displayed by Masson's staining (Figure 1a, 1b).

### UUO mice exhibited renal Klotho deficiency and renal injury

**Figure 2:**
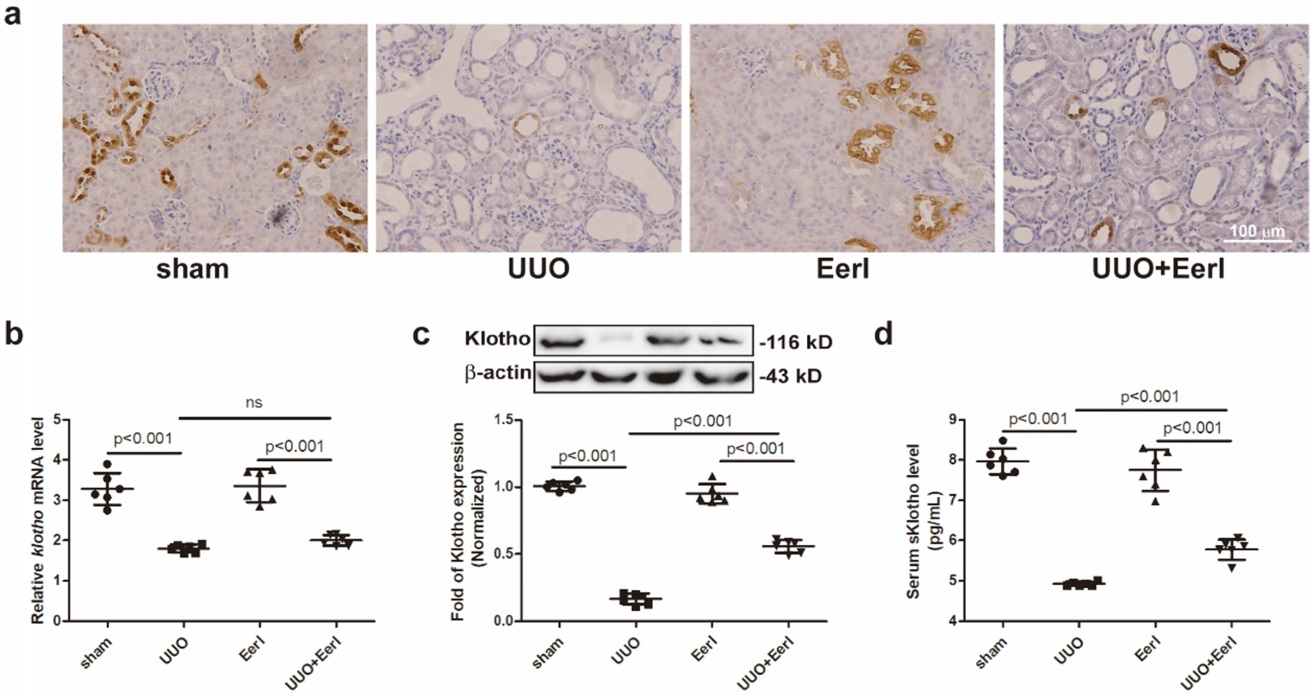
** EerI administration represses UUO-induced Klotho protein loss in mice.** (a) Representative immunohistochemical staining image of Klotho expression (scale bar, 100 μm). (b) PCR analysis of Klotho in renal tissue and their relative expression levels. (c) Western blotting analysis of Klotho in renal tissue and their relative expression levels. (d)Serum sKlotho levels in mice detected by ELISA. Data are presented as the mean ± SD (n = 6).

Klotho expressions in renal tissue and serum were separately determined. Unsurprisingly, Klotho was expressed mainly in distal tubules of sham group mice, while systematic Klotho deficiency was observed in UUO mice (Figure 2a, 2b, 2c and 2d). Compared with sham mice, a significant reduction in renal Klotho expression at protein or mRNA level and a moderate reduction in sKlotho levels were measured in UUO mice (Figure 2b-2d). Notably, Klotho deficiency correlated closely with renal histopathological injury and RIF.

### UUO promoted UPR and stimulated the ERAD pathway in fibrotic kidney

**Figure 3:**
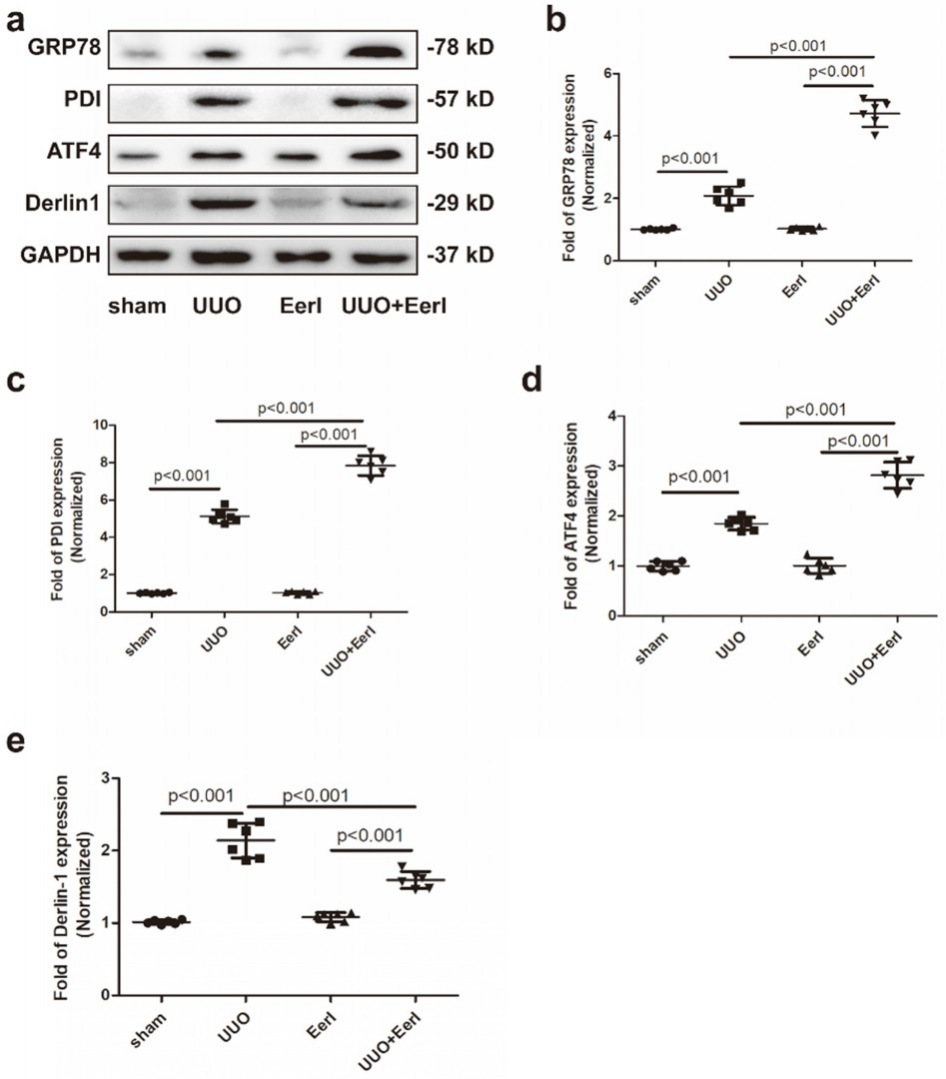
** EerI administration promoted-ERAD and stimulated-UPR pathway in fibrotic kidney.** (a) Representative western blotting images of GRP78, PDI, ATF4 and Derlin-1 expressions. The fold expression of GRP78 (b), PDI (c), ATF4(d), Derlin 1 (e). Data are presented as the mean ± SD (n = 6).

UUO-induced sustained stress promotes misfolded or unfolded protein accumulation and ignites ER stress 29, 30. As a consequence, UPR and ERAD may be invoked to preserve ER homeostasis. We observed UUO-induced upregulation of three UPR biomarkers, glucose-regulated protein 78 (GRP78), activating transcription factor 4 (ATF4) and protein disulfide isomerase (PDI), indicating that the UPR was stimulated (Figure 3a-3d). GRP78 is an ER-resident chaperone that can be released from three UPR sensors in response to retention of unfolded or misfolded proteins in the ER: inositol-requiring kinase 1α (IRE1α), protein kinase RNA-like ER kinase (PERK), and activating transcription factor 6 (ATF6). Subsequently, GRP78 activates downstream signaling involving IRE1α, PERK, and ATF6 31. The three UPR branches work together to alleviate ER stress either by stimulating proper protein folding or decreasing protein synthesis 32, 33. PDI, another chaperone involved in proper folding of newly synthesized proteins, acts by forming disulfide bonds 34. ATF4 serves as downstream signaling involving PERK and is to fold proteins properly by elevating chaperones^35^, ^36^. Derlin-1, a critical component of the ERAD pathway, retrotranslocates protein substrates from the ER to the cytosol for proteasomal degradation 37. In this study, Derlin-1 expression was dramatically elevated in UUO mice, suggesting that UUO contributed to activation of the ERAD pathway (Figure 3a, 3e). These results reveal that UUO-induced Klotho deficiency was associated with increased Klotho protein degradation.

### Inhibition of ERAD upregulated Klotho protein expression and attenuated kidney injury

To investigate the relationship between ERAD and Klotho deficiency, a specific inhibitor of ERAD - EerI, was administered to UUO mice 38, 39. Given that unfolded or misfolded Klotho proteins undergo ERAD in the cytoplasm and this part of immature Klotho cannot be expressed in the cell membrane, the immature Klotho has no impact on sKlotho expression. Because sKlotho (the functional isoform of Klotho) is generated from the cleaved extracellular domain of mature Klotho located in the membrane, an increase in sKlotho indicates the upregulation of functional Klotho. We demonstrated that EerI upregulates renal Klotho protein expression, as well as sKlotho levels (Figure 2a, 2c, 2d), without significantly altering Klotho mRNA level (Figure 2b). Interestingly, increased Klotho level generates kidney protective effects. In addition, this upregulation was accompanied by elevated expression levels of GRP78, PDI and ATF4 (Figure 3a-3d), indicating that UPR was ulteriorly enhanced in the presence of ERAD inhibition. Therefore, the increase of Klotho expression induced by ERAD-specific suppression was associated with enhanced UPR, which possibly strived to perform extra folding or maturation of Klotho to increase Klotho production under this condition.

### EerI abrogates TGF-β1-triggered Klotho reduction in cultured HK-2 cells

**Figure 4:**
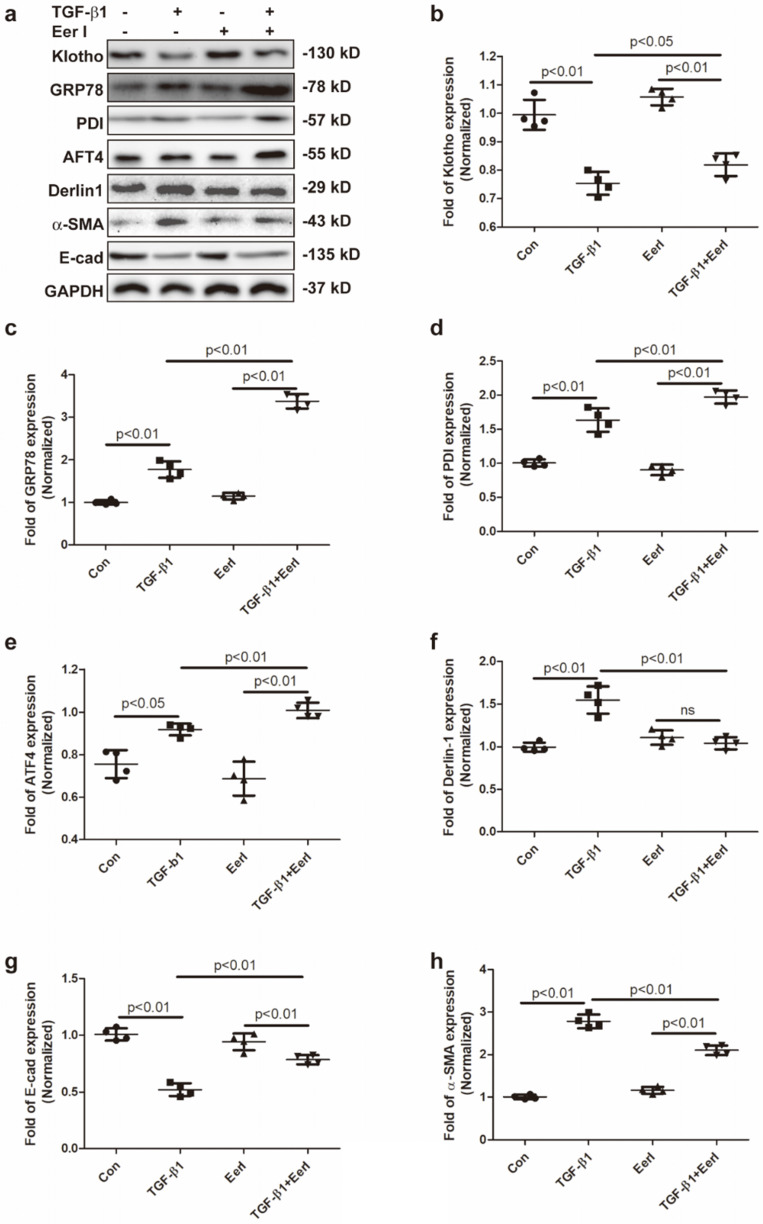
** EerI abrogates TGF-β1-triggered Klotho reduction in cultured HK-2 cells.** (a) Representative western blotting images of Klotho, GRP78, PDI, ATF4, Derlin-1, α-SMA, E-cad expressions. The fold expression of Klotho (b),GRP78 (c), PDI (d), ATF4 (e), Derlin-1 (f), α-SMA(g), E-cad(h) expressions. Data are presented as the mean ± SD (n = 6). ns, no significance.

TGF-β1 is known to be one of the main drivers in the pathogenesis and progression of RIF. Thus, the impact of ERAD on Klotho expression was also evaluated in TGF-β1-induced renal fibrosis in HK-2 cells. We observed that TGF-β1 stimulated upregulation of α-SMA and accompanied by downregulation of E-Cad (Figure 4a,4g,4h), indicating that TGF-β1 initiated renal fibrosis via epithelial mesenchymal transition (EMT) in HK-2 cells, which was in accordance with previous reports 40, 41. Concomitantly, TGF-β1 remarkably reduced Klotho expression, while increased GRP78, Derlin-1, ATF4 and PDI expressions in HK-2 cells (Figure 4a-4f). Interestingly, EerI treatment reversed Klotho expression at protein level and ameliorated EMT in TGF-β1-treated HK-2 cells (Figure 4a, 4b, 4g, 4h). Most importantly, Klotho restoration by EerI was accompanied by further upregulation of GRP78, PDI and ATF4 expressions compared with TGF-β1-treatment alone (Figure 4a, 4c-4e). These similar changes were in consistence with the observations from fibrotic kidney in UUO mice.

## Discussion

Herein, we demonstrated that RIF exhibited downregulation of Klotho protein and upregulation of UPR and ERAD components. ERAD-specific inhibitor elevated Klotho protein expression, without altering Klotho mRNA level, resulting in enhanced UPR. The current results strongly suggest that Klotho downregulation is linked to ERAD, and Klotho elevation by ERAD inhibition may be related to enhanced UPR and subsequent Klotho protein maturation. Thus, ERAD is implicated in the regulation of Klotho expression.

Sustained stresses, including inflammation, oxidative stress, hypoxia and ischemia, contribute to the accumulation of misfolded or unfolded proteins in the ER, which disrupt ER homeostasis 42. As a protective response, ER stress is consequently activated to restore ER homeostasis by coordinating proper folding of abnormal proteins through UPR or degrading of unfolded or misfolded proteins through ERAD 23, 42. During this process, GRP78 acts as chaperone and upstream modulator of three branches of the UPR (PERK, IRE1α and ATF6 pathways), and activates UPR by disassociating from PERK, IRE1α, and ATF6 43. PDI, an abundant oxidoreductase chaperone in ER, is required for proper protein folding 34 and catalyzes the formation of protein disulfide bonds and rearrangements during UPR 44. ATF4 is a DNA binding transcription factor and plays crucial roles in various physiological activities 45. As an important branch of UPRs, ATF4 can be activated by phosphorylation of eukaryotic initiation factor 2a (eIF2a) involving PERK signaling when ER homeostasis is disturbed. ATF4 activation aims to directly inhibit overall production of protein or enhance the proper folding of protein by regulating expressions of downstream ER and UPR related genes 35, 36. Consequently, ER or cellular homeostasis can be maintained under the diseased condition. Derlin-1 is mainly located in the ER, whereby it serves as an important component of the ERAD complex 37. Derlin-1 works in a complex with hydroxymethylglutaryl reductase degradation protein 1 and p97 to retrotranslocate unfolded or misfolded proteins from the ER lumen to the cytosol 26. Retrotranslocated proteins are recognized and subsequently degraded in the cytosol by the ubiquitin-proteasome system. Hence, UPR and ERAD synergistically restore cell homeostasis under sustained ER stress 46.

CKD is characterized by elevated inflammation, oxidative stress, and ischemia, which are persistent ER stress inducers that inevitably result in extra accumulation of misfolded or unfolded proteins in the ER. ER stress, including UPR and ERAD, are subsequently initiated to address the abnormally retained proteins 17, 47. Indeed, expressions of ER stress- and UPR-related genes are significantly increased and closely related with kidney function in CKD patients 18. Moreover, this observation has been confirmed in a number of preclinical studies 20, 27, 48-50. Therefore, UPR and ERAD are implicated in the pathogenesis of kidney diseases including CKD, emerging as novel targets for CKD management 26-28, 51, 52.

Klotho protein has critical functions and displays diverse kidney-protective effects 13, 53. We previously reported that Klotho ameliorated RIF by suppressing epithelial-mesenchymal or endothelial-mesenchymal transitions 1, 54. Unsurprisingly, Klotho deficiency is associated with increased RIF and promotes CKD development 10, 55, 56. In this study, we observed that UUO induced a significant decrease of systematic Klotho expression and deteriorated kidney injury; however, upregulation of Klotho expression was associated with amelioration of kidney injury. This observation was also demonstrated in HK-2 cells exposed to TGF-β1. Thus, strategies to upregulate Klotho expression or block Klotho downregulation may be applicable for attenuating RIF and delaying CKD progression 12, 57. However, the molecular mechanism underlying Klotho deficiency in kidney diseases is still not yet fully understood, leading to intensive ongoing research in this area. Previous studies suggest that regulation of Klotho expression in CKD involves a variety of mechanisms, such as epigenetic and non-epigenetic disorders, that reportedly suppress Klotho gene transcripts to contribute to Klotho downregulation 14. Yet, few studies emphasized whether a lack of Klotho protein was attributed to its accelerated degradation. ERAD represents a principal quality control machinery by which misfolded or unfolded proteins are cleared to maintain ER homeostasis 23. However, whether ERAD was linked to Klotho deficiency in kidney remained largely unknown.

A recent study by Delitsikou et al. demonstrated an association between ER stress activation and reduced Klotho protein levels, and specific inhibition of ER stress restored Klotho protein expression without changing *klotho* mRNA levels in a proteinuria CKD model 21. This study was the first to reveal that enhanced Klotho degradation was associated with a loss of Klotho expression, but the exact mechanism remained to be elucidated. CKD is characterized by persistent ER stress that yields increased accumulation of misfolded or unassembled proteins (including Klotho) in the ER. Because Klotho contains eight putative N-linked glycosylation sites, it is presumably subjected to post-translational modifications before maturation 5. As an important compensatory mechanism, ERAD is activated to alleviate protein overload and maintain normal cell function in the context of persistent ER stress. Given this, we hypothesize that Klotho may be a potential substrate of ERAD, such that activated ERAD accelerates nascent or immature Klotho protein degradation to cause a deficiency of Klotho protein. Accordingly, we observed reduced Klotho expression accompanied by enhanced Derlin-1 levels in fibrotic kidney or TGF-β1-treated HK-2 cells, suggesting that ERAD was enhanced and possibly involved in the degradation of Klotho protein in RIF. Furthermore, increased GRP78, PDI and ATF4 expression levels in the UUO group indicated that upregulation of UPR possibly compensated for extra proper folding of proteins.

Intriguingly, the ERAD-specific inhibitor EerI abrogated ERAD-induced Klotho loss and rescued Klotho expression in fibrotic kidney and HK-2 cell treated by TGF-β1. Notably, the change of Klotho expression occurred mainly at its protein level, not its transcript level. These observations strongly indicate that increased degradation of Klotho contributed to Klotho deficiency, and Klotho may be a possible ERAD substrate. Mechanistically, ERAD inhibition resulted in further accumulation of misfolded proteins, including nonfunctional nascent Klotho precursor protein, in the ER. To distinguish mature from immature Klotho, we measured plasma levels of sKlotho because misfolded Klotho protein precursor (immature form) cannot be translocated from the ER or located in the plasma 58. sKlotho is primarily yielded by cleavage of the extracellular domain of mature Klotho, which is expressed in the cell membrane. Thus, sKlotho represents the functional Klotho isoform and level of mature Klotho. The extra deposition of misfolded protein elicited by ERAD inhibition further stimulates UPR to enhance cellular protein-folding capacity and generate more functional protein 46. This phenomenon was in accordance with upregulated expression levels of GRP78, PDI and ATF4. The above findings indicate a novel regulatory mechanism of Klotho expression exerted by ERAD inhibition, which may be UPR-dependent; although, the exact mechanism underlying the interaction among Klotho protein, ERAD, and UPR remains to be elucidated.

This study has several limitations. Firstly, traditional indicators of kidney function were not measured. Instead, renal pathological lesions were assessed. However, impairments of kidney function do not always parallel with histopathological changes. This indicates the effect of ERAD inhibition on kidney function is not determined to some extent. Secondly, we only evaluated the short-term impact of ERAD inhibition on Klotho level and ER homeostasis, thus, the long-term impact of ERAD inhibition was still to be investigated in future. Thirdly, UPR and ER stress constituent a complex regulatory network when ER homeostasis is disordered under pathological conditions. That's to say, in addition to PERK- eIF2α-ATF4 signaling, other UPR pathways including IRE1α and ATF6 should be detected as well.

In summary, we demonstrated the involvement of ERAD in modulation of Klotho expression and ascribed the underlying mechanism to potential enhancements of the UPR. Thus, ERAD and UPR may represent novel regulatory mechanisms and interventional targets for rescuing Klotho expression under the condition of Klotho-deficient states.
